# Conditional deletion of MAD2B in forebrain neurons enhances hippocampus-dependent learning and memory in mice

**DOI:** 10.3389/fncel.2022.956029

**Published:** 2022-09-23

**Authors:** Li Cheng, Yanfang Su, Kaining Zhi, Yaru Xie, Chun Zhang, Xianfang Meng

**Affiliations:** ^1^Department of Neurobiology, Institute of Brain Research, School of Basic Medical Sciences, Tongji Medical College, Huazhong University of Science and Technology, Wuhan, China; ^2^Department of Nephrology, Union Hospital, Tongji Medical College, Huazhong University of Science and Technology, Wuhan, China

**Keywords:** *MAD2B*, learning and memory, dendritic spine, synapse, hippocampus

## Abstract

Mitotic arrest deficient 2-like protein 2 (MAD2B) is not only a DNA damage repair agent but also a cell cycle regulator that is widely expressed in the hippocampus and the cerebral cortex. However, the functions of MAD2B in hippocampal and cerebral cortical neurons are poorly understood. In this study, we crossed *MAD*2*B*^flox/flox^ and calcium/calmodulin-dependent protein kinase II alpha (*Camk2a*)*-Cre* mice to conditionally knock out *MAD2B* in the forebrain pyramidal neurons by the Cre/loxP recombinase system. First, RNA sequencing suggested that the differentially expressed genes in the hippocampus and the cerebral cortex between the WT and the *MAD2B* cKO mice were related to learning and memory. Then, the results of behavioral tests, including the Morris water maze test, the novel object recognition test, and the contextual fear conditioning experiment, suggested that the learning and memory abilities of the *MAD2B* cKO mice had improved. Moreover, conditional knockout of *MAD2B* increased the number of neurons without affecting the number of glial cells in the hippocampal CA1 and the cerebral cortex. At the same time, the number of doublecortin-positive (DCX^+^) cells was increased in the dentate gyrus (DG) of the *MAD2B* cKO mice. In addition, as shown by Golgi staining, the *MAD2B* cKO mice had more mushroom-like and long-like spines than the WT mice. Transmission electron microscopy (TEM) revealed that spine synapses increased and shaft synapses decreased in the CA1 of the *MAD2B* cKO mice. Taken together, our findings indicated that MAD2B plays an essential role in regulating learning and memory.

## Introduction

Learning and memory are vital foundations of life activities. Neurodegenerative diseases such as Alzheimer's disease (AD) and Parkinson's disease (PD), which are accompanied by a gradual decline in learning and memory ability, are increasing every year. AD is the most common neurodegeneration disease with eventual memory decline (Hu et al., 2016). Some neuronal pathological changes, including neuronal loss (Spangenberg et al., 2016), synapse loss (Duncan and Valenzuela, 2017), and decrements in dendritic arborization and spine density (Maiti et al., 2021), occur in AD. Abnormal gene expression in some brain areas can also lead to changes in learning and memory ability (Konopka et al., 2010; Brault et al., 2021; Kar et al., 2021). For example, Bin1 deficiency in neurons leads to the impairment of spatial learning and memory due to abnormal presynaptic regulation (De Rossi et al., 2020). In mice, the brain-specific knockout of follistatin results in declines in spatial learning and working memory, as well as deficits in LTP and neurogenesis in the hippocampus (Chen et al., 2021).

Mitotic arrest deficient 2-like protein 2 (MAD2B, also known as MAD2L2 or REV7) is a component of the mitotic spindle assembly checkpoint, which plays an important role in regulating cell cycle, gene transcription, DNA repair, and carcinogenesis. It has been reported that MAD2B is mainly expressed in neuronal-like cells with pyramidal shapes in the hippocampus and cerebral cortex (Meng et al., 2012). In addition, MAD2B is related to the pathophysiology of diabetic encephalopathy (Meng et al., 2014). Under high glucose treatment, primary cultured neurons with high MAD2B expression undergo apoptosis. MAD2B is also overexpressed in the cerebral cortex of diabetic rats. As one of the risk factors for AD (Baglietto-Vargas et al., 2016; Khan et al., 2021; Zhang et al., 2021; Athanasaki et al., 2022), diabetes encephalopathy shares similar pathogenesis with AD. In diabetes encephalopathy, the proliferation and neuronal differentiation of newborn cells in the dentate gyrus (DG) and neuronal density in the hilar region (Beauquis et al., 2009) is reduced. Moreover, diabetic rats have low dendritic spine density and downregulated synaptic-related protein expression in the hippocampus (Jin et al., 2018). Transmission electron microscope indicates that pathological changes in synaptic ultrastructure such as the thickness of postsynaptic density and the synaptic gap of the hippocampal CA1 fields as well as decreased survival of CA1 neurons occur in diabetes encephalopathy (Xu et al., 2021). These abnormal changes in synaptic plasticity have become the main reasons for the decline in learning and memory in diabetes encephalopathy. Although MAD2B is closely related to diabetes encephalopathy (Meng et al., 2014), its direct role in the nervous system remains to be clarified.

The conditional knockout (cKO) of a gene in specific neurons has become a valuable method for studying gene function. The calcium/calmodulin-dependent protein kinase II alpha (*Camk2a*)*-Cre* mouse line, in which forebrain pyramidal neurons in postnatal mice are targeted (Tsien et al., 1996; Wang et al., 2021a), is widely used in this field. Therefore, in the present study, we generated the *MAD2B* cKO mice under the control of *Camk2a* gene regulatory elements by using the Cre/loxP system and investigated the direct role of the *MAD2B* gene in the nervous system. We first analyzed the RNA sequencing (RNA-seq) results of the hippocampus and the cerebral cortex of the *MAD2B* cKO mice and found several differentially expressed genes (DEGs) that might be involved in learning and memory regulation. Next, we conducted behavioral tests on learning and memory. The *MAD2B* cKO mice demonstrated enhanced hippocampus-dependent learning and memory in the Morris water maze (MWM) test, the novel object recognition test (NORT), and the contextual fear conditioning (CFC) experiment. Finally, we found that the number of doublecortin-positive (DCX^+^) cells in the DG, mushroom-like spines, and spine synapses in the CA1 region increased. These findings provide a foundation for revealing the role of MAD2B in the central nervous system and for treating cognitive dysfunction.

## Materials and methods

### Animals

All procedures for the care and use of laboratory animals were approved by the Institutional Guidelines and Animal Care and Use Committee of Huazhong University of Science and Technology, Wuhan, China. In addition, animal experiments were performed in accordance with the Guidelines for the Care and Use of Laboratory Animals from the National Institutes of Health, USA. Mice were housed in a 21 ± 1 °C temperature room with constant humidity under a 12 h light/dark cycle (8:00 AM−8:00 PM). Food and water were freely available.

All male mice (7–8 weeks) shared the same C57/BL6J genetic background (Jackson Laboratory stock). *MAD2B* was conditionally knocked out in the forebrain pyramidal neurons under the control of the *Camk2a* promoter with the Cre/loxP system. *MAD2B*^*flox*/*flox*^ mice, which have loxP sites flanking exons 3 and 4 of the *MAD2B* gene, were generated and *Camk2a Cre* mice were purchased from Shanghai Model Organisms Center, Inc. *MAD2B*^*flox*/*flox*^ and *Camk2a-Cre* mice were intercrossed to generate *MAD2B*^*flox*/*flox*^; *Camk2a Cre*^+^ mice (cKO mice) and *MAD2B*^*flox*/*flox*^; *Camk2a Cre*^−^ mice (WT mice) as previously described (McGill et al., 2018).

All mice were handled by the experimenter for a week before the behavioral experiments to reduce the unnecessary influence of stress. All mice were acclimated to the behavioral room for 2 h before experiments. All behavioral experiments were conducted between 8:00 AM−8:00 PM with dim light and performed in a double-blinded manner.

### Genotyping

Mice were genotyped through polymerase chain reaction (PCR) by using tail DNA as previously described (Sadick et al., 2022). In brief, a 3 mm piece of tissue was cut from the end of the tail of a 1-month-old mouse and then digested in 75 μl of alkaline lysis regent (25 mM NaOH and 0.2 mM EDTA in DNase- and RNase-free water) at 98 °C for 1 h. Subsequently, the mixture was centrifuged for 1 min at 1,000 rpm, and 75 μl of neutralizing reagent (40 mM Tris-HCl, pH 5.5) in DNase- and RNase-free water) was added to each sample on ice. Then, the solution was centrifuged at 4,000 rpm for 3 min at 4 °C, and the supernatant containing genomic DNA (gDNA) was collected for later genotyping. The PCR system comprised 10 μl of 2× Taq Plus Master Mix (Vazyme, code number: P212); 0.5 μl of forward primer (10 pmol/μl); 0.5 μl of reverse primer (10 pmol/μl); and 9 μl of gDNA. The cycling reaction was step1: 94 °C for 3 min; step 2 (35 repeats): 94 °C for 30 s, 58 °C for 30 s, and 72 °C for 30 s; step 3: 72 °C for 5 min; and step 4: holding at 12 °C. The primer sequences were *MAD2B* forward: 5′-TCTTCCCTTAGATTGGGTTTCTC-3′ and *MAD2B* reverse: 5′-GCACGAATAGGACAAACAGCAA-3′ and *Camk2a-Cre* forward: 5′-GGGGAGGTAGGAAGAGCGATGA-3′ and *Camk2a-Cre* reverse: 5′-ATCGACCGGTAATGCAG-3′.

### Open field test

The open field test was performed as previously described (Yoshizaki et al., 2020). Briefly, each mouse was placed in the center of an open-field apparatus (50 × 50 × 50 cm with four black walls and a white floor) and allowed to move freely in the apparatus, which was divided into 25 small squares. The nine middle squares were defined as the central zone, and the four squares in four corners were defined as the corner zone. Behaviors were automatically recorded using a camera connected to a digital tracking device (Xinruan Information Technology Co. Ltd, Shanghai, China) for 10 min. Once finished, the mouse was moved back to its home cage immediately. The equipment was cleaned with 70% ethanol between mice. A computer with the Super maze software (Xinruan Information Technology Co. Ltd, Shanghai, China) was then used to process the tracking information. Data on total moving distance and speed and the time in the corner zone or central zone were then analyzed.

### Elevated plus maze

The elevated plus maze was 50 cm in length, 10 cm in width, and 40 cm in height with two open arms, two closed arms, and a central zone. The elevated plus maze test was performed as previously described (Qi et al., 2022). Briefly, each mouse was placed in the central zone facing the open arm, and its behaviors were recorded for 10 min. Once finished, the mouse was moved back to its home cage immediately. The equipment was cleaned with 70% ethanol between mice. Behaviors were automatically recorded by a camera connected to a digital tracking device (Xinruan Information Technology Co. Ltd, Shanghai, China). A computer with the Super maze software (Xinruan Information Technology Co. Ltd, Shanghai, China) then processed the tracking information. The time that the mice spent in the open arms and the closed arms was then analyzed.

### Morris water maze

The MWM was performed as previously described (Qu et al., 2021). The setup for MWM consisted of a circular pool (diameter of 1.2 m and height of 50 cm) filled with water that was maintained at 24–25 °C and whitened with titanium dioxide. The maze was located in a room with numerous extra-maze visual cues and divided into four equal quadrants. A platform with a 10 cm diameter was placed in a fixed quadrant (target quadrant) of the pool. On the first day, the platform was placed 1–2 cm above the water surface (Bromley-Brits et al., 2011; Luo et al., 2020) to enable the mice to see the platform and cues. The influence of mouse vision on the experimental results was thus excluded. Each mouse was trained for three trials with a 30 min inter-trial interval. From the second to the 5th day, each mouse was trained for three trials to find the platform that was hidden 1–2 cm below the water surface. The mouse was placed into the pool from the other three quadrants in each trial, except for the target quadrant, and given 60 s to find the platform. If the mouse failed to find the platform within 60 s, it was guided to the platform by the experimenter. The mouse was allowed to stay on the platform for 20 s. On the 6th day (probe test), the mouse was placed in the pool from the opposite side of the target quadrant for 60 s. Once finished, the mouse was moved back to its home cage immediately. Behaviors were automatically recorded by a camera (fixed to the device 1 m from the water surface) connected to a digital tracking device (Xinruan Information Technology Co. Ltd, Shanghai, China). A computer with the Morris water maze software (Xinruan Information Technology Co. Ltd, Shanghai, China) then processed the tracking information. Escape latency, escape length on training days, velocity, the number of platform crossings, and total distance traveled in the target quadrant on the probe test day was automatically recorded for later analysis.

### Novel object recognition test

The NORT was conducted in an open field apparatus (50 × 50 × 50 cm) with four black walls and a white floor as previously described (Li et al., 2021). Briefly, the mice were acclimated to the conditions of the experimental room for 2 h. Then, each mouse was presented with two identical objects (familiar objects; 3 × 3 × 6 cm blue plastic cuboids) placed at the rear left and right corners (fixed onto the floor with adhesive tape so that the mouse cannot push them down) in the open field apparatus. The mouse was allowed to explore the objects freely for 5 min. The apparatus and objects were thoroughly cleaned with 70% ethanol between mice. One h later, one of the familiar objects was randomly replaced with a new object (a 3 × 3 × 7 cm red wooden object with a mushroom-like shape), and the mouse was again placed in the open field for 5 min. Once finished, the mouse was moved back to its home cage immediately. The apparatus and objects were thoroughly cleaned with 70% ethanol between mice. Behaviors were automatically recorded by a camera connected to a digital tracking device (Xinruan Information Technology Co. Ltd, Shanghai, China). A computer with the Super maze software (Xinruan Information Technology Co. Ltd, Shanghai, China) then processed the tracking information. The time mice spent exploring each object (within 2 cm of the object) with their noses or paws was calculated. The discrimination index (DI) was defined as the percentage of time spent exploring the novel object to the total time spent exploring both objects.

### Contextual fear conditioning

The CFC was performed as previously described (Zhao et al., 2019). On the training day, each mouse was acclimated to the fear conditioning chamber for 5 min. Then, the mouse received four trials of electric foot shock (0.5 mA, 2 s duration) with a 1 min intertrial interval and 1 min of no stimulation followed by the last foot shock. Once finished, the mouse was moved back to its home cage immediately. Twenty-four h later, the mouse was placed in the same chamber for 5 min. The equipment was cleaned with absolute ethanol between mice. Behaviors were automatically recorded by a camera connected to a digital tracking device (Xinruan Information Technology Co. Ltd, Shanghai, China). A computer with the fear conditioning software (Xinruan Information Technology Co. Ltd, Shanghai, China) then processed the tracking information. The freezing time was recorded and analyzed. Freezing behavior was defined as immobility for more than 2 s.

### Rotarod test

The rotarod test was carried out with a rotarod machine (YLS-4C, Yiyan, Jinan, China) as previously described (Genc et al., 2021). Before the test, each mouse was habituated to staying on a stationary rod for 2 min. Then, each mouse was placed on the machine rotating from 5 to 40 rpm for 5 min and tested for three trials with a 30-min interval between each trial. Once falling, the mouse was moved back to its home cage immediately, and the latency of the mouse falling was automatically recorded by the machine. If the mouse insisted on the apparatus for more than 5 min of a trial, the latency was counted as 5 min, and the mouse was then moved from the apparatus. The equipment was cleaned with 70% ethanol between mice. The average latency of three trials represented the motor function of a mouse.

### Immunofluorescence

Mice were sacrificed immediately after the last behavioral task. Immunofluorescence was performed as previously described (Jiang et al., 2021). Briefly, after fixation and dehydration, brains were sliced into 30 μm coronal sections by using a cryostat microtome. The brain slices immediately adhered to the slides. Then, brain slices at the same level were randomly selected and subjected to the following procedures: Membranes were lysed with 0.5% Triton-X for 10 min. Subsequently, non-specific protein binding sites were blocked with 5% donkey serum for 1 h at room temperature. The slices were then incubated overnight at 4 °C with mouse monoclonal anti-neuron-specific nuclear protein (NeuN) (1:100, MAB377, Millipore), rabbit polyclonal anti-glial fibrillary acidic protein (GFAP) (1:200, 16825-1-AP, Proteintech), rabbit monoclonal anti-induction of brown adipocytes 1 (Iba1) (1:100, ab178847, Abcam), rabbit polyclonal anti-doublecortin (DCX) (1:200, 13925-1-AP, Proteintech), and rabbit polyclonal anti-MAD2B (1:200 ab180579, Abcam) antibodies diluted with 5% bovine serum albumin (BSA). After being rinsed three times for 5 min each with PBS, the brain slices were incubated with fluorochrome-conjugated secondary antibodies (1:200, DyLight-488-labeled or DyLight-594-labeled donkey anti-mouse or donkey anti-rabbit, Jackson, USA) for 1 h at room temperature in the dark. Then, the brain slices were incubated with Hoechst 33258 (Thermo Fisher Scientific, Shanghai, China) for 5 min. Finally, the brain slides were automatically scanned by a fluorescence microscope (Olympus, SV120, Japan) for imaging. The VS-ASW-S6 software was used to acquire the images of MAD2B, NeuN, GFAP, and Iba1. A Carl Zeiss LSM780 laser scanning confocal microscope (Zeiss Microsystems, Jena, Germany) was used to acquire images of DCX. The number of NeuN-, GFAP-, and Iba1-positive cells were determined in a 100 × 100 μm square and the number of DCX^+^ cells was obtained from the DG zone in each brain slice. All positive puncta were counted by Fiji software (National Institutes of Health, Bethesda, MD, USA). The relative number of positive cells was normalized to that in the WT group by the following formula: the relative number of positive cells of each mouse = the number of positive cells of each mouse/the average number of positive cells in the WT group.

### Protein extraction and Western blot

Western blot was conducted as previously described (Zhong et al., 2020). According to the references (McGill et al., 2018), the whole hippocampal and cerebral cortical tissues were lysed with RIPA buffer (Beyotime Biotechnology, Shanghai, China) supplemented with protease and phosphatase inhibitors (Thermo Fisher Scientific, Shanghai, China). Protein quantification was performed by using a BCA Protein Assay Kit (Beyotime Biotechnology, Shanghai, China). Lysates were then boiled with 5× loading buffer (Service Biotechnology Co., Ltd., Wuhan) for 8 min at 98 °C. A total of 50 μg of protein per sample was separated through SDS-polyacrylamide gel electrophoresis and transferred to PVDF membranes (Bio-Rad Laboratories). After blocking in 5% skimmed milk for 1 h at room temperature, the membranes were incubated with MAD2B (rabbit, 1:1000, Abcam, ab180579) and α-tubulin (mouse, 1:30000, Proteintech, 66031-1-AP) antibodies overnight at 4 °C and then incubated with rabbit peroxidase-conjugated secondary antibody for 1 h at room temperature. Specific signals were detected with film through ECL (Epizyme Biotech, Shanghai, China). The results were obtained by normalizing the intensity of each target band to its corresponding α-tubulin band with Fiji software (National Institutes of Health, Bethesda, MD, USA).

### RNA-seq analysis

RNA-seq was performed as previously described (Koenen et al., 2018). Briefly, RNA samples from the whole hippocampal and cerebral cortical tissues according to the references (McGill et al., 2018) were extracted following the RNeasy Mini kit (Qiagen, 74104) instructions. Complementary DNA (cDNA) library construction and sequencing were conducted by the Beijing Genomics Institute (BGI) by using the BGISEQ-500 platform. The data obtained through sequencing were called raw reads or raw data, which were submitted to NCBI (the accession number was PRJNA866907). The raw reads were subjected to quality control (QC) to determine whether the sequencing data were suitable for subsequent analysis. After quality control, the filtered clean reads were compared with the reference sequence. After the comparison, whether the comparison results had passed the second QC (QC of alignment) was determined by counting the comparison rate and the distribution of reads on the reference sequence. If they passed, gene quantitative analyses (principal component analysis, correlation, and differential gene screening) were performed on the basis of gene expression level, and the DEGs in the screened samples were subjected to Gene Ontology (GO) analyses, Kyoto Encyclopedia of Genes and Genomes (KEGG) pathway enrichment analyses, and other in-depth mining analyses. Each gene was normalized to the maximum fragments per kilobase of transcript per million reads mapped (FPKM) between the two groups. The DEGs between the WT and *MAD2B* cKO groups were screened out through the DESeq2 method (Love et al., 2014). Thus, the relative expression number was between 0 and 1. The significant differences in genes, pathways, and terms were corrected through the Bonferroni method based on a Q value ≤ 0.05. GraphPad Prism 8 was used to reanalyze the relative expression, which was presented in the form of a gene expression heatmap.

### Total RNA extraction and quantitative PCR

Total RNA was extracted from the whole hippocampal and cerebral cortical tissues according to the references (McGill et al., 2018; Zhao et al., 2020) by using TriZol reagent (Invitrogen, Shanghai, China) and chloroform. The reverse transcription of total RNA (1,000 ng) into cDNA was performed with a SYBR Premix Ex Taq TM Kit (Takara, Kyoto, Japan). Quantitative PCR was performed with a Fast Start Universal SYBR Green Master (ROX) PCR kit (Vazyme, Q311-02, Nanjing, China). Genes were selected from the DEGs based on their relevance to learning and memory. The selected genes included activating transcription factor 4 (*Atf4*), *Camk2a*, cortistatin (*Cort*), RAB3B, member RAS oncogene family (*Rab3b*), and methylenetetrahydrofolate reductase (*Mthfr*). The expression levels of *MAD2B, Atf4, Camk2a, Cort, Rab3b*, and *Mthfr* were determined by using the following primers synthesized by Sangon Biotech (Sangon Biotech Co. Ltd., Shanghai, China): *MAD2B*: Forward-−5′ATTCTCTATGTGCGCGAGGTC-3′; Reverse-−5′TCCAGGAGAGGTTTGACGCA-3′; *Atf4*: Forward-−5′AGTTTAGAGCTAGGCAGTGAAG-3′; Reverse-−5′CATACAGATGCCACTGTCATTG-3′; *Camk2a*: Forward-−5′GCACCACTACCTTATCTTCGAT-3′; Reverse-−5′TGGCATCAGCTTCACTGTAATA-3′; *Cort*: Forward-−5′CCTTCTGACTTTCCTTGCCT-3′; Reverse-−5′GAAGTTCTTGCAGGGCTTTTTA-3′; *Rab3b*: Forward-−5′TTCTCGTGGGAAATAAGTGTGA-3′; Reverse-−5′GAGTCAGACATCTTATCGCAGA-3′; *Mthfr*: Forward-−5′GAAACCATCCTGCATATGACCT-3′; Reverse-−5′CAAAATAGTCAGCAAACTCGGT-3′. All cDNA analyses were performed in triplicate by using a 7500 qPCR instrument (Applied Biosystems). The results were normalized to the housekeeping mouse gene β*-actin* (Sangon Biotech Co. Ltd., Shanghai, China) with primers as followed: β*-actin*: Forward-−5′GGACTCCTATGTGGGTGACGAG-3′; Reverse-−5′TCACGGTTGGCCTTAGGGTT-3′.

### Golgi staining and Sholl analysis

Golgi staining was performed with the FD Rapid Golgi Stain Kit (FD Neurotechnologies, Columbia, SC, USA) by the instructions previously described (Wang et al., 2021b). An upright microscope (Nikon Ni-E; Japan) with a 10× objective lens or a 100× oil objective lens was used for image collection. NIS-Elements software was used to acquire images. The results were analyzed by Fiji software (National Institutes of Health, Bethesda, MD, USA). For each selected sample, the analyzed branch length was at least 10 μm. The dendritic spine density was evaluated as the number of spines per 10 μm of the dendritic branch. For Sholl analysis, neurons in CA1 were manually traced by the Neuron J plugin. The intersections of sholl rings (centered on the cell body and starting radius of 10 μm with diameter increments of 10 μm) and dendrites were analyzed by the Sholl plugin.

### Transmission electron microscopy

The CA1 region was cut into 1 mm^3^ section within 1–3 min, and the stained cuprum grids were obtained as previously described (Rybka et al., 2019). Briefly, the samples were transferred into fresh transmission electron microscopy (TEM) fixative (Service Biotechnology Co., Ltd., Wuhan) at 4 °C for preservation. The tissues were washed with 0.1 M PB (pH 7.4) three times for 15 min each. Then, the CA1 tissues were postfixed with 1% OsO_4_ (Ted Pella Inc.) in 0.1 M PB (pH 7.4) for 2 h at room temperature. After OsO_4_ was removed, the tissues were rinsed with 0.1 M PB (pH 7.4) three times for 15 min each time. The tissues were dehydrated with ethanol (30, 50, 70, 80, 95, and 100%) (Sinopharm Group Chemical Reagent Co. LTD) for 20 min each time than with acetone (Sinopharm Group Chemical Reagent Co. LTD) two times for 15 min each time at room temperature. The tissues were permeabilized in a mixture of acetone and EMBed 812 (acetone: EMBed = 1:1 and 1:2) and pure EMBed 812 (SPI, 90529-77-4) before being embedded in pure EMBed 812 in a 37 °C oven overnight. The embedding models with the resin and the samples were placed in a 65 °C oven for more than 48 h for polymerization. Then, the resin blocks were removed from the embedding models and stored at room temperature for future use. Next, the resin blocks were cut into 60–80 nm thick slices on an ultramicrotome (Leica, Leica UC7), and the tissues were fished out onto 150 mesh cuprum grids with formvar film. Then, the cuprum grids were stained and finally imaged through TEM (HITACHI, HT7800) at 80 kV. Images were acquired under 5,000 × or 20,000 × magnification. The number of synapses per optical field was calculated by 29 optical fields from each group to quantify synapses.

### Statistical analysis

All the results are displayed as the means ± standard error of the mean (s.e.m), with ^*^p < 0.05, ^**^p < 0.01, ^***^p < 0.001, and ^****^p < 0.0001. Here, “ns” indicates no significance. Unpaired two-tailed Student's *t*-tests with or without Welch's correction were performed. Multiple-*t*-tests with Bonferroni–Dunn correction were performed on MWM learning days (escape latency and length). Two-way repeated-measures ANOVA was performed on the fear acquisition stage and Sholl analysis. All statistical analyses were performed by using GraphPad Prism 8 software.

## Results

### The generation and identification of the *MAD2B* cKO mice

**Figure 1:**
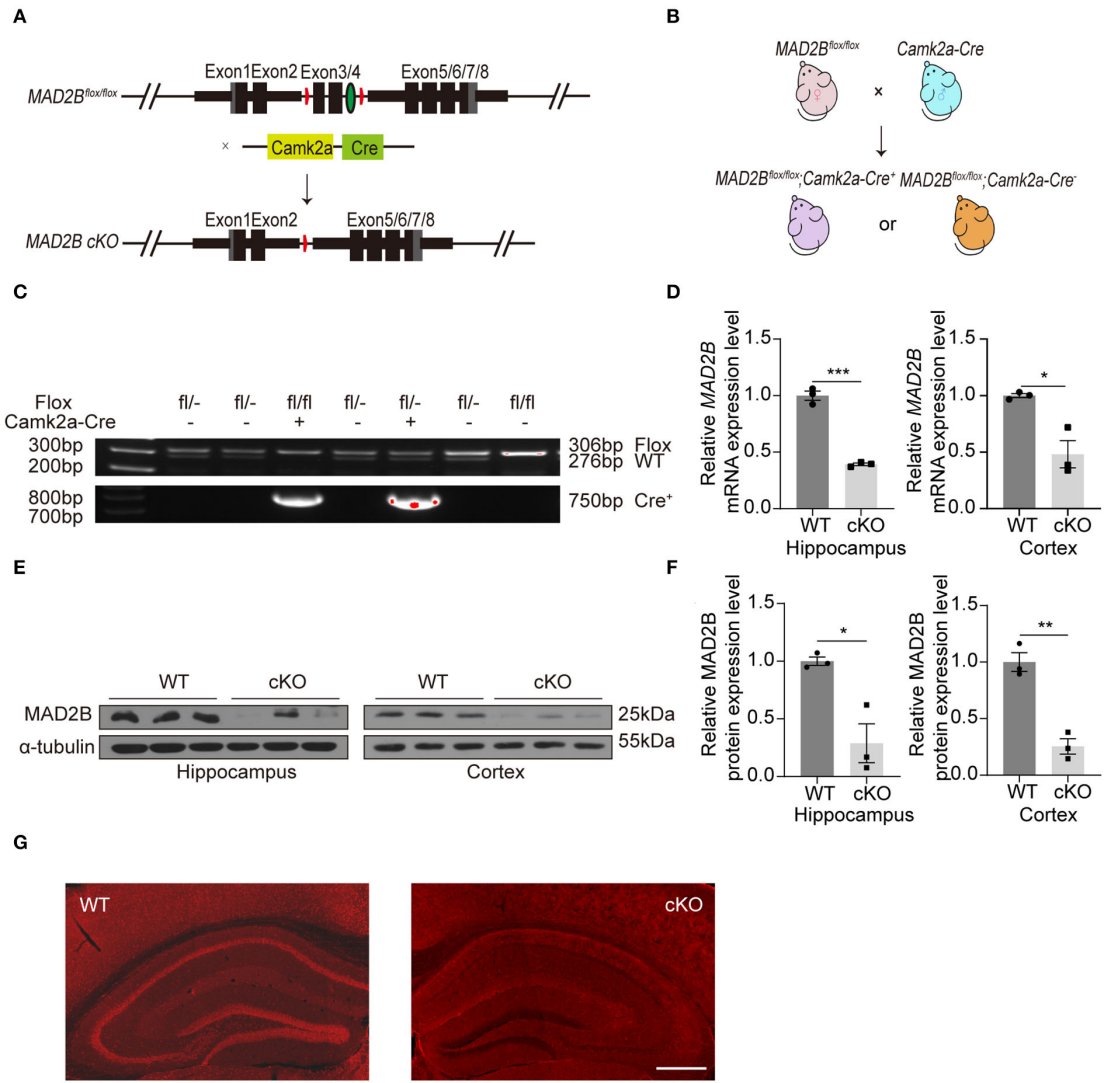
The generation and identification of the *MAD2B* cKO mice. **(A)** Schematic diagram of *MAD2B* cKO by the Cre/loxP system. **(B)** Schematic representation of the breeding strategy. **(C)** Tail DNA PCR confirmation of transgenic *MAD2B* cKO mice. **(D)** qRT-PCR validation of the downregulation of the *MAD2B* gene in the hippocampus (*p* = 0.0001, *t* = 14.310, unpaired two-tailed *t-*test) and cerebral cortex (*p* = 0.0467, *t* = 4.272, unpaired two-tailed *t-*test with Welch's correction), *n* = 3. **(E,F)** Western blot analysis showing the downregulation of MAD2B protein expression in the hippocampus (*p* = 0.0459, *t* = 4.136) and cerebral cortex (*p* = 0.0023, *t* = 6.943), *n* = 3. Unpaired two-tailed *t-*test. **(G)** Representative immunofluorescence images validating the cKO of MAD2B in the hippocampus and the cerebral cortex including MPtA, LPtA, PtPD, PtPR, and S1BF, the scale bar is 500 μm. *p < 0.05, **p < 0.01, ***p < 0.001. All data are displayed as means ± s.e.m.

A transgenic mouse line expressing a floxed allele of *MAD2B* was established to study the role of *MAD2B* in learning and memory. Mice with *MAD2B* cKO in *Camk2a*-specific forebrain pyramidal neurons were generated by using the Cre/loxP recombination system (Figure 1A). We crossed *MAD2B*^*flox*/*flox*^ mice with *Camk2a Cre* mice to generate the needed mice for experiments (Figure 1B). Mice genotypes for the *Cre* and floxed alleles were confirmed by performing PCR on gDNA from the mice tails (Figure 1C). We used qRT-PCR (Figure 1D hippocampus, *p* = 0.0001, *t* = 14.310, Figure 1D cortex, *p* = 0.0467, *t* = 4.271) and Western blot analyses (Figures 1E,F, hippocampus *p* = 0.0459, *t* = 4.136, cortex *p* = 0.0023, *t* = 6.943) to confirm the cKO of MAD2B in the hippocampus and the cerebral cortex. The expression of MAD2B was significantly reduced at the mRNA and protein levels in the hippocampus and the cerebral cortex of the *MAD2B* cKO mice. Immunofluorescence of the brain slices also showed a significant deficiency of MAD2B in the CA1, DG, CA3, and cerebral cortex including medical parietal association cortex (MPtA), lateral parietal association cortex (LPtA), parietal cortex, posterior area, dorsal part (PtPD), parietal cortex, posterior area, rostral (PtPR), and primary somatosensory cortex, barrel field (S1BF; Figure 1G). However, there still existed residual expression of MAD2B at the mRNA and protein level, as well as positive puncta in the immunofluorescence image, which might be due to the lack of Cre recombinase expression.

### Altered signaling pathways and expression profiles of downstream genes in the hippocampus and the cerebral cortex of the *MAD2B* cKO mice

**Figure 2:**
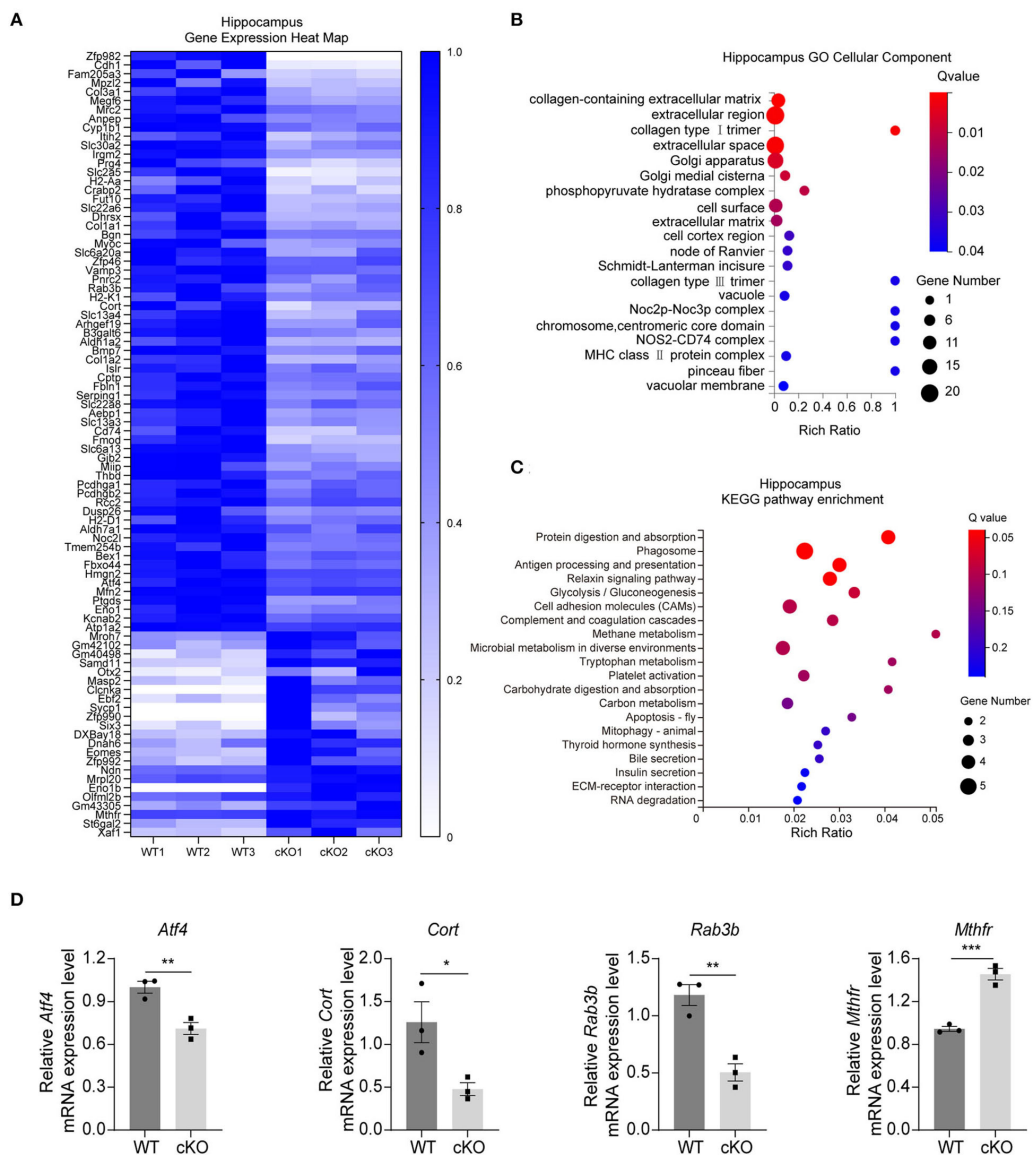
Changes in signaling pathways and expression profiles of genes in the hippocampus of the *MAD2B* cKO mice. **(A)** Heat map of DEGs in the hippocampus; the deeper the purple, the higher the expression. **(B)** GO analysis of cellular components of DEGs in the hippocampus, *n* = 3, *p* < 0.05. The size of the bubble indicates the gene number, the color depth indicates the Q-value, and the rich ratio indicates the gene number/the total gene number in the y-axis item. **(C)** KEGG pathway enrichment analysis of DEGs in the hippocampus, *n* = 3, *p* < 0.05. The size of the bubble indicates the gene number, the color depth indicates the Q-value, and the rich ratio indicates the gene number/the total gene number in the y-axis item. **(D)** qRT-PCR identification of DEGs in the hippocampus (*Atf4 p* = 0.0079, *t* = 4.973; *Cort p* = 0.0354, *t* = 3.125; *Rab3b p* = 0.0046, *t* = 5.712; and *Mthfr p* = 0.0009, *t* = 8.831, unpaired two-tailed *t-*test) *n* = 3, *p < 0.05, **p < 0.01, ***p < 0.001. All data are displayed as means ± s.e.m.

We first performed RNA-seq analyses to understand the further effects of *MAD2B* cKO. We identified 89 DEGs in the hippocampus, among which 24 were upregulated and 65 were downregulated. The results were displayed in the form of a heatmap (Figure 2A, *q* value < 0.05, |log2FC| > 0). Next, the DEGs were subjected to GO analysis and were divided into three categories, including biological processes, cellular components, and molecular functions. The changed cellular components included extracellular parts (collagen-containing extracellular matrix, extracellular region, and extracellular space) and intracellular parts (Golgi apparatus, Golgi medial cisterna, and phosphor–pyruvate hydratase complex) (Figure 2B). These cellular components regulate synaptic plasticity structurally and functionally (Stawarski et al., 2014; Wójtowicz et al., 2015; Yang et al., 2022). In the KEGG pathway enrichment analysis (*p* < 0.05 Figure 2C) between the WT and cKO groups, the biological processes mainly included protein metabolism (protein digestion and absorption), glucose metabolism (glycolysis/gluconeogenesis, carbohydrate digestion, and absorption), and other types of metabolism (methane, tryptophan, and carbon) and hormone regulation (thyroid hormone synthesis). These biological processes directly or indirectly regulate learning and memory (Knezovic et al., 2018; Chaalal et al., 2019; Ajoy et al., 2021; Zhang et al., 2022). Moreover, three downregulated DEGs (*Atf4 p* = 0.0079, *t* = 4.973; *Cort p* = 0.0354, *t* = 3.125; and *Rab3b p* = 0.0046, *t* = 5.712) and one upregulated DEG (*Mthfr p* = 0.0009, *t* = 8.831) (Figure 2D) were confirmed by qRT-PCR. These four genes were selected because of their relationship with learning and memory (Tallent et al., 2005; Tsetsenis et al., 2011; Jadavji et al., 2015; Amar et al., 2021).

**Figure 3:**
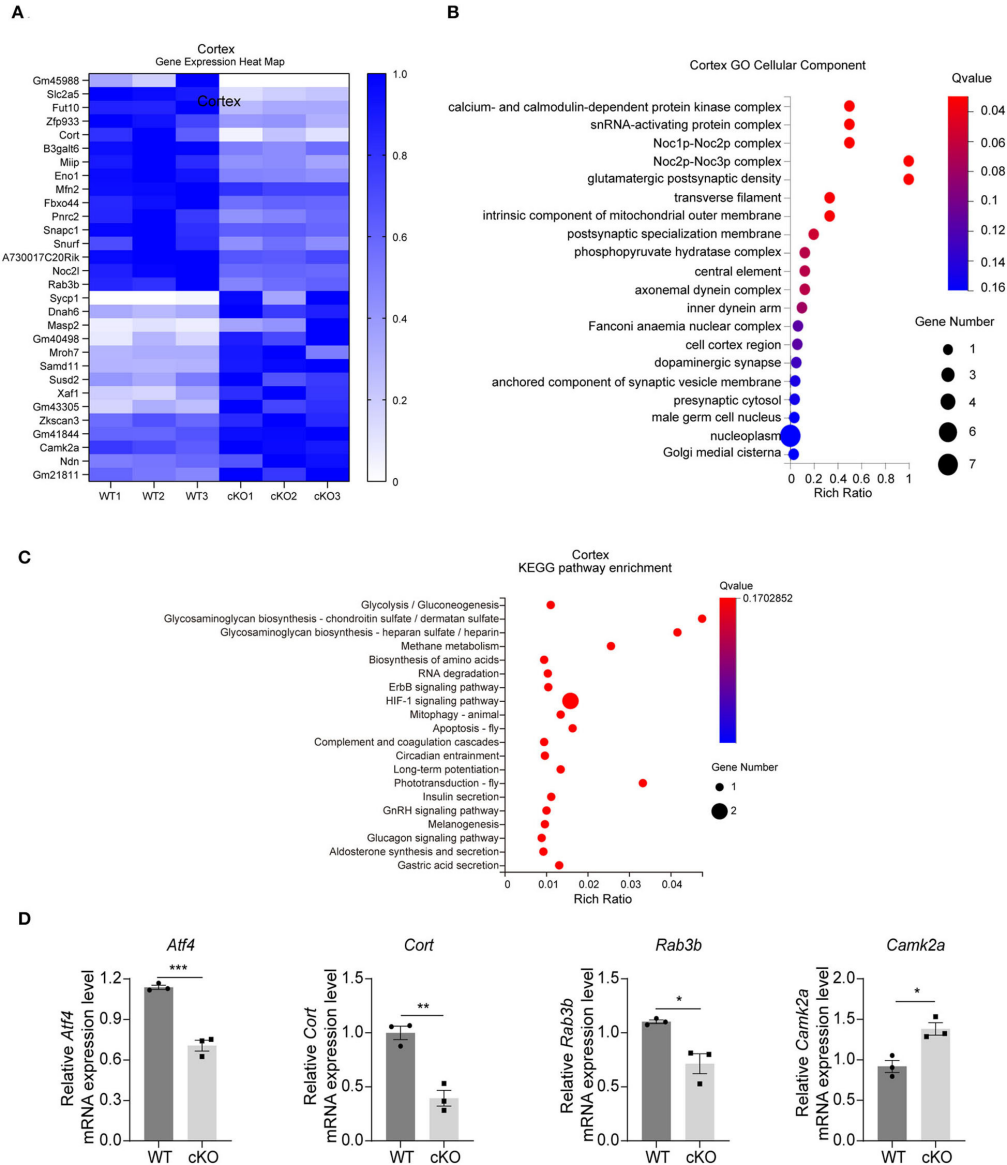
Changes in signaling pathways and expression profiles of genes in the cerebral cortex of the *MAD2B* cKO mice. **(A)** Heat map of DEGs in the cerebral cortex; the deeper the purple, the higher the expression. **(B)** GO analysis of cellular components in the cerebral cortex, *n* = 3, *p* < 0.05. The size of the bubble indicates the gene number, the color depth indicates the Q-value, and the rich ratio indicates the gene number/the total gene number in the y-axis item. **(C)** KEGG pathway enrichment analysis of DEGs in the cerebral cortex, *n* = 3, *p* < 0.05. The size of the bubble indicates the gene number, the color depth indicates the Q-value, and the rich ratio indicates the gene number/the total gene number in the y-axis item. **(D)** qRT-PCR identification of DEGs in the cerebral cortex (*Atf4 p* = 0.0006, *t* = 9.942; *Cort p* = 0.0032, *t* = 6.322; *Rab3b p* = 0.0148, *t* = 4.106; and *Camk2a p* = 0.0123, *t* = 4.334), *n* = 3, *p < 0.05, **p < 0.01, ***p < 0.001. All data are displayed as means ± s.e.m.

The RNA-seq results in the cerebral cortex indicated that there were 30 DEGs between the WT and the *MAD2B* cKO mice. Among these DEGs, 14 were upregulated and 16 were downregulated relative to those in the WT mice. The results were displayed in the form of a heatmap (Figure 3A, *q* value < 0.05, |log2FC| > 0). Next, the DEGs were subjected to GO analysis, and cellular components were displayed (Figure 3B). In contrast to that of the hippocampus, the GO cell component analysis of the cerebral cortex showed that the functions of DEGs could be mainly divided into the following aspects: axon (axonemal dynein complex), presynaptic (presynaptic cytosol), synaptic vesicle (anchored component of synaptic vesicle membrane), and postsynaptic (glutamatergic postsynaptic density, and postsynaptic specialization membrane), undoubtedly suggesting that these DEGs are closely related to synaptic functions. Although cortical KEGG suggested that the change in the main pathway after *MAD2B* cKO was related to glucose metabolism, several other signaling pathways were found to be involved in the regulation of learning and memory. These pathways included the relaxin signaling pathway, the long-term potentiation (LTP), the ErbB signaling pathway, and the HIF1 signaling pathway (Figure 3C). Similar to those in the hippocampus, three downregulated DEGs (*Atf4 p* = 0.0006, *t* = 9.942; *Cort p* = 0.0032, *t* = 6.322; and *Rab3b p* = 0.0148, *t* = 4.106) and one upregulated DEG (*Camk2a p* = 0.0123, *t* = 4.334), which could promote learning and memory processes (Lisman et al., 2012), were confirmed through qRT-PCR (Figure 3D).

Taken together, the GO and KEGG pathway analyses of the DEGs in the hippocampus and the cerebral cortex suggested that *MAD2B* cKO in Camk2a-positive neurons affected the expression levels of genes related to learning and memory.

### Improved learning and memory in the *MAD2B* cKO mice

**Figure 4:**
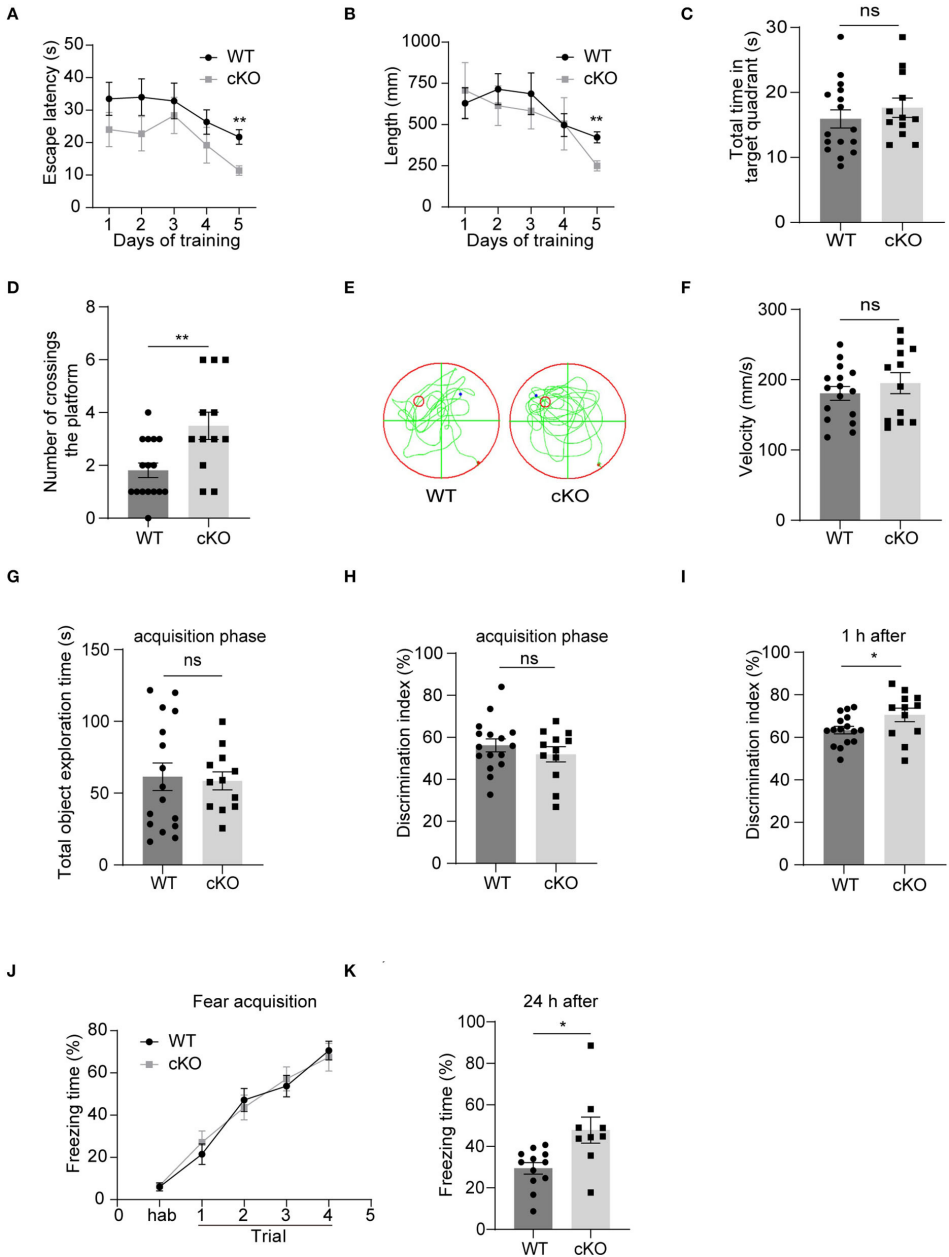
The *MAD2B* cKO mice showed increased hippocampus-dependent learning and memory abilities. **(A)** Escape latency during training days in MWM (multiple-*t*-test, *p* = 0.22 on day1, *p* = 0.17 on day2, *p* = 0.58 on day3, *p* = 0.27 on day4, *p* = 0.001 on day5). **(B)** Escape length during training days in MWM (multiple-*t*-test, *p* = 0.67 on day1, *P* = 0.51 on day2, *p* = 0.56 on day3, *p* = 0.97 on day4, *p* = 0.001 on day5). **(C)** Total time in the target quadrant on the probe test day (*p* = 0.4077, *t* = 0.8415, unpaired two-tailed *t*-test). **(D,E)** Times of crossing the platform on the probe test day in MWM (*p* = 0.0048, *t* = 3.082, unpaired two-tailed *t*-test). **(F)** Swimming velocity on the probe test day in MWM (*p* = 0.4040, *t* = 0.8484, unpaired two-tailed *t*-test). **(G,H)** Total object exploration time (*p* = 0.8198, *t* = 0.2301) and DI (*p* = 0.3724, *t* = 0.9076) of the familiar stage in NORT, unpaired two-tailed *t*-tests. **(I)** DI in the recognition stage in NORT (*p* = 0.0468 *t* = 2.087, unpaired two-tailed *t*-test. *n* = 16 in the WT group and *n* = 12 in the cKO group). **(J)** Freezing of the adaption time and the learning curve of fear acquisition for the CFC test (two-way ANOVA with Sidak's multiple comparisons test, interaction: *F*_(4,76)_ = 0.4746, *p* = 0.7543, DF = 4; trials: *F*_(2.949,56.04)_ = 73.44, *p* < 0.0001, DF = 4; genotype: *F*_(1,19)_ = 0.01582, *p* = 0.9012, DF = 1). **(K)** Freezing time in the CFC test 24 h after the fear acquisition, *n* = 12 in the WT group, *n* = 9 in the cKO group, *p* = 0.0212, Welch-corrected *t* = 2.684, unpaired *t*-test with Welch's correction, *p < 0.05, **p < 0.01, ns indicates no significance. All data are displayed as means ± s.e.m.

We explored hippocampal-associated learning and memory in the *MAD2B* cKO mice. First, we used the MWM to detect spatial learning and memory abilities. The results showed that on the 5th day of the training period, the escape latency (Figure 4A, multiple-*t*-test, *p* = 0.22 on day 1, *p* = 0.17 on day 2, *p* = 0.58 on day 3, *p* = 0.27 on day 4, *p* = 0.001 on day 5) and swimming length (Figure 4B, multiple-*t*-test, *p* = 0.67 on day 1, *p* = 0.51 on day 2, *p* = 0.56 on day 3, *p* = 0.97 on day 4, *p* = 0.001 on day 5) of the cKO mice were less than the WT mice. On the probe test day, although the *MAD2B* cKO mice traveled for a similar time in the target quadrant to the WT mice (Figure 4C, *p* = 0.4077, *t* = 0.8415), they crossed the location of the missing platform more frequently than the WT mice (Figures 4D,E, *p* = 0.0048, *t* = 3.082). The swimming velocity on the probe test day did not significantly differ between the WT and the cKO mice (Figure 4F, *p* = 0.4040, *t* = 0.8484).

Then, we performed the NORT, which detected short-term memory. First, the mice were familiarized with two identical objects. We found no significant difference in the total exploration time (Figure 4G, *p* = 0.8198, *t* = 0.2301) and DI between the two groups (Figure 4H, *p* = 0.3724, *t* = 0.9076), indicating that the mice had no preference for similar objects in different locations. However, when one of the two familiar objects was replaced with a new object 1 h after the acquisition phase, the *MAD2B* cKO mice were more inclined to approach the new object than the WT mice given that the *MAD2B* cKO mice showed higher DI than the WT mice (Figure 4I, *p* = 0.0468, *t* = 2.087).

We used CFC to detect the effects of *MAD2B* cKO on the fear acquisition and fear memory. The *MAD2B* cKO and the WT groups exhibited identical intact post-shock freezing during fear acquisition (Figure 4J, two-way ANOVA with Sidak's multiple comparisons test, interaction: *F*_(4,76)_ = 0.4746, *p* = 0.7543, DF = 4; trials: *F*_(2.949,56.04)_ = 73.44, *p* < 0.0001, DF = 4; genotype: *F*_(1,19)_ = 0.01582, *p* = 0.9012, DF = 1), which the mice learned to link the chamber environment to the foot shock, indicating that fear acquisition was unaffected by *MAD2B* cKO. However, when the mice were placed in the fear conditioning chamber after 24 h, the *MAD2B* cKO mice exhibited a considerably longer freezing time than the WT mice (Figure 4K, *p* = 0.0212, *t* = 2.684).

Anxiety and locomotion ability can affect learning and memory ability (Csernansky et al., 2005; Ueda et al., 2017; Mett et al., 2021). Therefore, we performed the open field, the elevated plus maze, and the rotarod test to examine the influence of *MAD2B* cKO on anxiety behavior and locomotion. Total moving distance (Supplementary Figure 1A *p* = 0.5979, *t* = 0.5340) and speed (Supplementary Figure 1B, *p* = 0.3712, *t* = 0.9100) in the open field test showed no significant difference in exploration and locomotion between the two groups. The time mice spent in the corner zone (Supplementary Figure 1C, *p* = 0.3481, *t* = 0.9555) or central zone (Supplementary Figure 1D, *p* = 0.2191, *t* = 1.259) in the open field test and the time mice spent in open arms (Supplementary Figure 1E, *p* = 0.2727, *t* = 1.121) or closed arms (Supplementary Figure 1F, *p* = 0.7198, *t* = 0.3657) in the elevated plus maze test showed that the anxiety levels in the *MAD2B* cKO group were comparable with those in the WT group. In addition, we evaluated the motor coordination abilities of the WT and the *MAD2B* cKO mice through the rotarod test. The results showed no difference in motor coordination abilities between the two groups (Supplementary Figure 1G *p* = 0.0787, *t* = 1.833).

### Increased number of neurons in the CA1 region and the cerebral cortex in the *MAD2B* cKO mice

**Figure 5:**
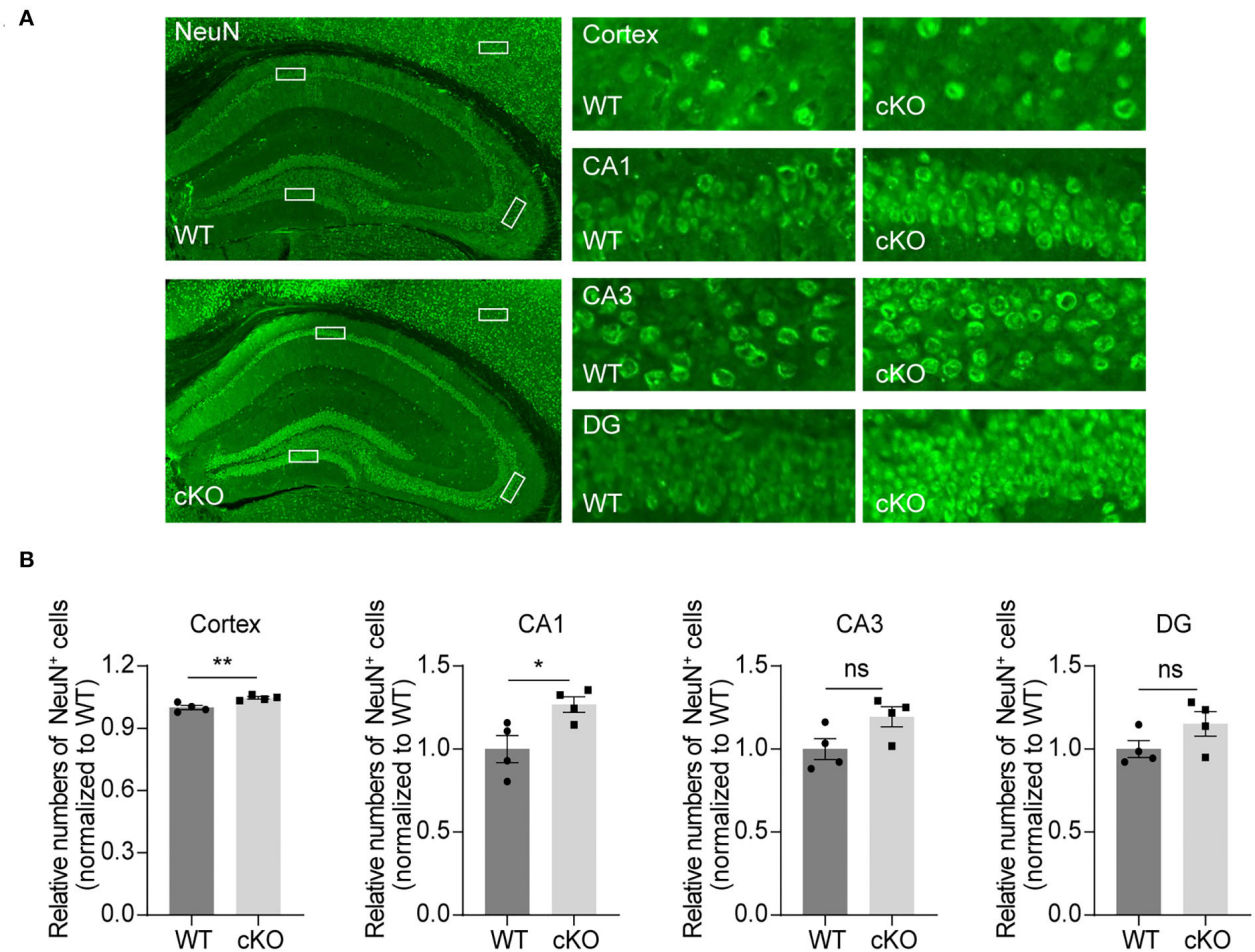
Increased neurons in the CA1 region and the cerebral cortex. **(A)** Representative fluorescent images of NeuN in the hippocampus and the cerebral cortex. The dashed box indicates the representative areas of the cerebral cortex, CA1, CA3, and DG. The scale bar is 500 μm. The representative enlarged images of these areas are shown on the right, and the scale bar is 50 μm. **(B)** Quantification of NeuN in the cerebral cortex (*p* = 0.0084, *t* = 3.85), CA1 (*p* = 0.0294, *t* = 2.845), CA3 (*p* = 0.0678, *t* = 2.224) and DG (*p* = 01413, *t* = 1.693), unpaired two-tailed *t*-tests, *n* = 4. *p < 0.05, **p < 0.01, ns indicates no significance. All data are displayed as means ± s.e.m.

To explore the effects of *MAD2B* on different kinds of cells in the hippocampus and cerebral cortex, we used NeuN, GFAP, and Iba1 to label neurons, astrocytes, and microglia. The results showed that the *MAD2B* cKO mice had more neurons in the cerebral cortex (*p* = 0.0084, *t* = 3.856) and the CA1 area (*p* = 0.0294, *t* = 2.845) than WT mice. However, the number of neurons in the CA3 (*p* = 0.0678, *t* = 2.224) and DG (*p* = 0.1413, *t* = 1.693) of the hippocampus (Figures 5A,B) did not differ between the two groups. No significant differences were found between the number of GFAP- and Iba1-positive cells in the hippocampus and the cerebral cortex of the WT and the *MAD2B* cKO mice (Supplementary Figures 2A–D, *p* > 0.05).

### *MAD2B* cKO promoted neurogenesis in the DG of the hippocampus

**Figure 6:**
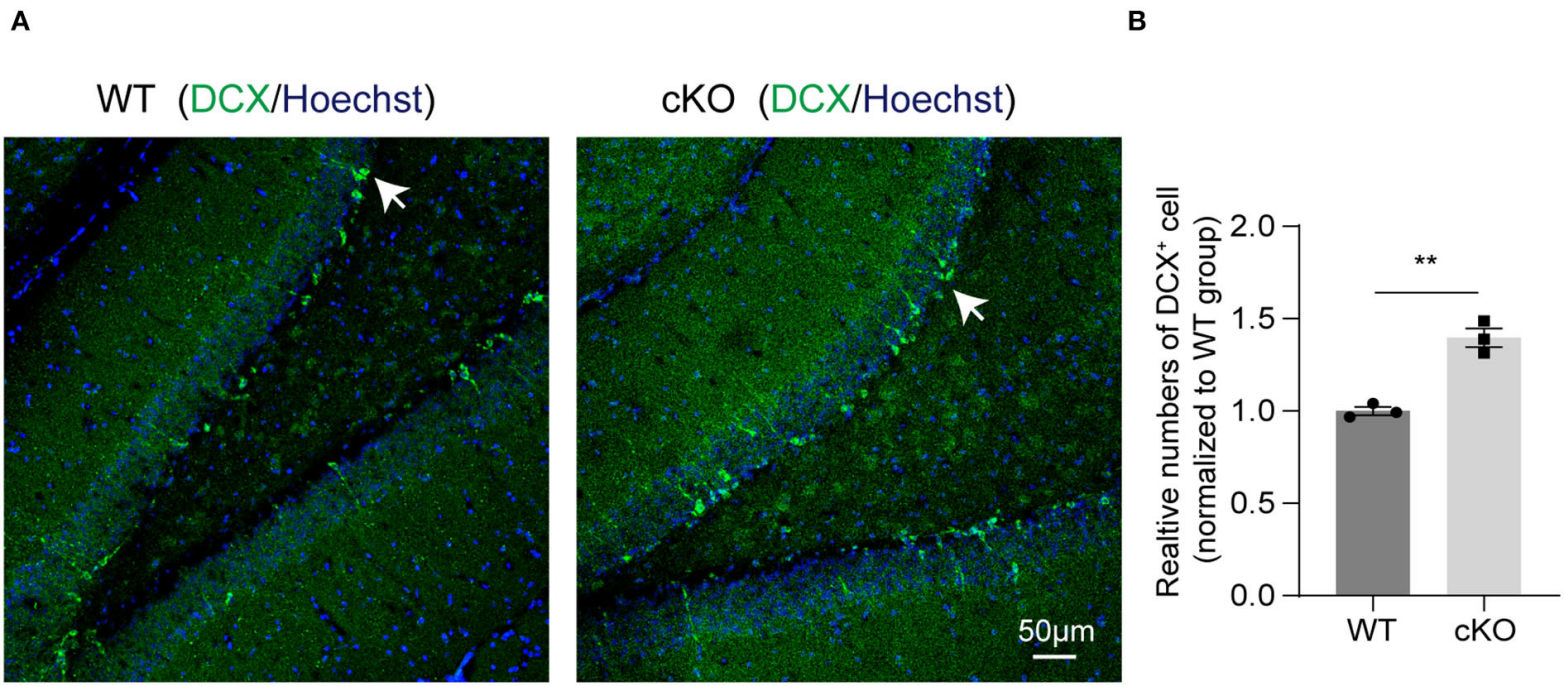
*MAD2B* cKO promoted neurogenesis in the DG of the hippocampus. **(A)** Representative confocal fluorescent images of DCX in the DG of the WT and the cKO groups. White arrows point to DCX positive neurons. The scale bar is 50 μm. **(B)** Quantification of DCX^+^ cells in the DG of the two groups (*n* = 3, *p* = 0.0019, *t* = 7.236, unpaired two-tailed *t*-test). **P < 0.01. All data are displayed as means ± s.e.m.

We next explored if *MAD2B* cKO influenced the levels of neurogenesis in the hippocampus. DCX, a developmentally regulated, microtubule-associated protein expressed in migrating and differentiating neurons, is now widely used as a marker of newly born neurons in the adult hippocampus. Our immunofluorescence result showed that *MAD2B* cKO increased the number of DCX^+^ cells in the DG (Figures 6A,B, *p* = 0.0019, *t* = 7.236) of the hippocampus.

### Increased dendritic spines and spine synapses in the CA1 area of the *MAD2B* cKO mice

**Figure 7:**
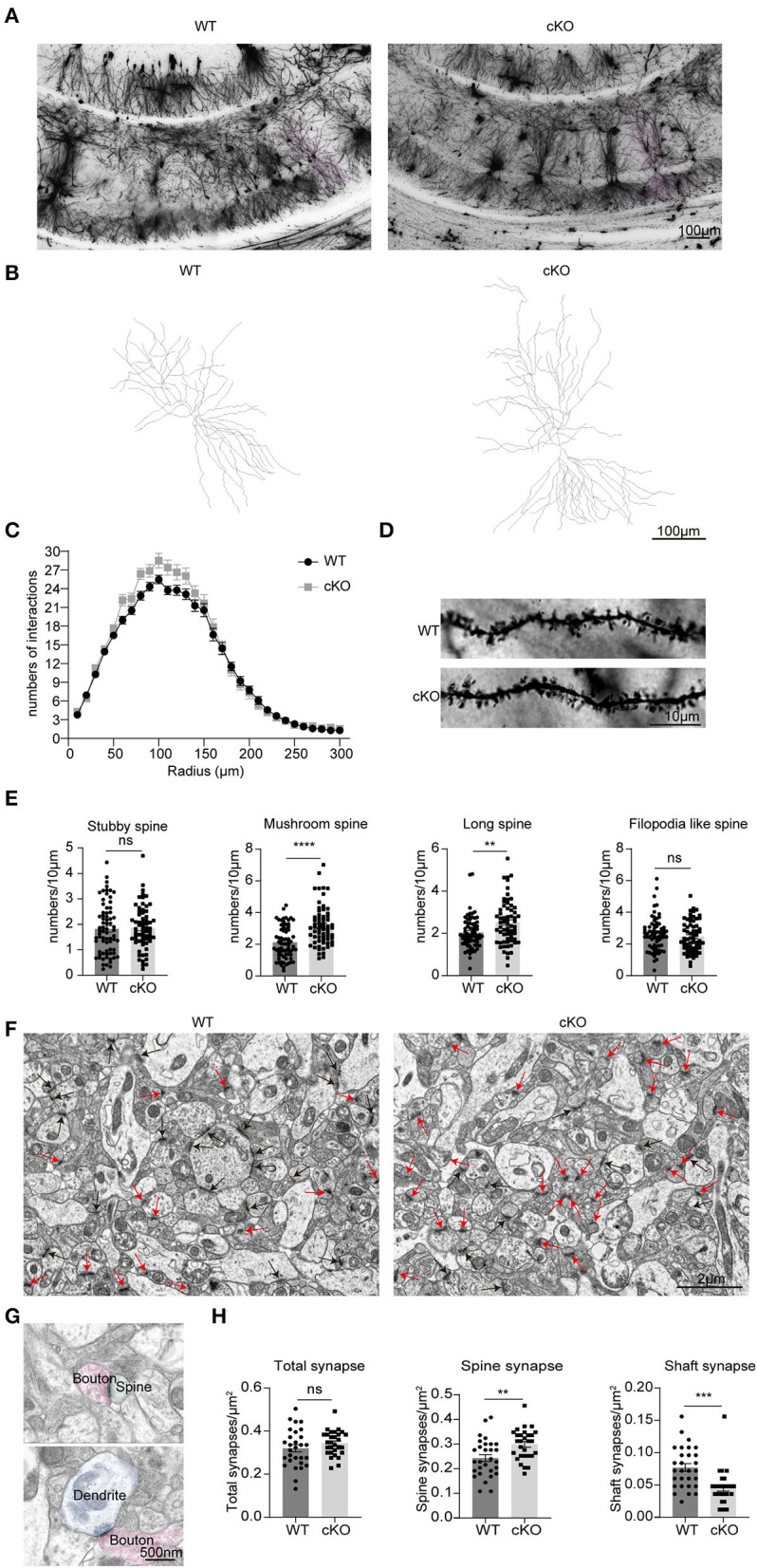
Changes in neuronal complexity, dendritic spines, and synapses of the *MAD2B* cKO mice. **(A)** Representative images of neurons in the CA1 region; the scale bar is 100 μm. **(B)** Representative traces of CA1 neurons; the scale bar is 100 μm. **(C)** The number of intersections in the Sholl analysis of pyramidal cells in the CA1 region (*n* = 50 neurons in the WT group and *n* = 35 neurons in the cKO group from three mice each, two-way RM ANOVA, interaction: *F*_(29,2436)_ = 2.724, *p* < 0.0001, DF = 29; radius: *F*_(4.761,399.9)_ = 460.7 *p* < 0.0001, DF = 29; genotypes: *F*_(1,84)_ = 2.429, *p* = 0.1229, DF = 1). **(D)** Representative images of dendritic spines; the scale bar is 10 μm. **(E)** Statistics of various types of dendritic spines, *n* = 71 dendritic branches in the WT group and *n* = 65 dendritic branches in the cKO group from three mice each, unpaired two-tailed *t*-tests with or without Welch's correction (stubby spine, *p* = 0.8031, *t* = 0.2519, mushroom-like spine, *p* < 0.0001, *t* = 5.319; long spine, *p* = 0.0027, *t* = 3.060; filopodia-like spine, *p* = 0.4089, t = 0.8285). **(F)** Representative TEM images of synapses in the CA1 region. The red arrows point to spine synapses and the black arrows point to shaft synapses. The scale bar is 2 μm. **(G)** The upper image shows spine synapse and lower image shows shaft synapse. The scale bar is 500 nm. **(H)** Quantification analyses of total synapses (*p* = 0.2242, Welch-corrected *t* = 1.231), spine synapses (*p* = 0.0035, *t* = 3.044), and shaft synapses (*p* = 0.0002, *t* = 4.028), unpaired two-tailed *t*-tests with or without Welch's correction. *n* = 29 field of views from three mice in each group. **p < 0.01, ***p < 0.001, **** *p* < 0.0001, ns indicates no significance. All data are displayed as means ± s.e.m.

We used Golgi–Cox-stained mice coronal sections cut at the level of the CA1 region (Figures 7A,B) to explore the effects of *MAD2B* cKO in dendritic branches and dendritic spines. The intersections of rings and dendrites were analyzed by Sholl rings at 10 μm-diameter increments centered on the cell body. Sholl analysis revealed that the cKO of *MAD2B* did not influence dendritic arborization in CA1 (Figure 7C, two-way RM ANOVA, interaction: *F*_(29,2436)_ = 2.724, *p* < 0.0001, DF = 29; radius: *F*_(4.761,399.9)_ = 460.7 *p* < 0.0001, DF = 29; genotypes: *F*_(1,84)_ = 2.429, *p* = 0.1229, DF = 1). Dendritic spines were visible under high magnifications of the dendritic arbor. We analyzed four different types of dendritic spines: stubby-, mushroom-, long-, and filopodia-like (Hering and Sheng, 2001). Our results showed that the *MAD2B* cKO mice exhibited considerably more mushroom-like (*p* < 0.0001, *t* = 5.319) and long-like dendritic spines (*p* = 0.0027, *t* = 3.060) than the WT mice and comparable numbers of stubby-like (*p* = 0.8015, *t* = 0.2519) and filopodia-like spines (*p* = 0.4089, *t* = 0.8285) as the WT mice (Figures 7D,E). In addition, we used TEM to observe synapses in the CA1 region. Here, we mainly observed spine synapses (excitatory synapses) (upper Figure 7G) and shaft synapses (inhibitory synapses) (lower Figure 7G). The results showed that the *MAD2B* cKO mice had more spine synapses (*p* = 0.0035, *t* = 3.044) and fewer shaft synapses (*p* = 0.0002, *t* = 4.028) than the WT mice. However, no significant difference in the total number of synapses (*p* = 0.2242, *t* = 1.231) was found between the two groups (Figures 7F–H).

## Discussion

In mammals, MAD2B is an inhibitor of the anaphase-promoting complex/cyclostome APC/Cdh1 (Reimann et al., 2001), which has a role in placental development and the ability of learning and memory, especially memory in CFC (Li et al., 2008). APC/Cdh1 is also an E3-ubiquitinated protein-degrading enzyme that degrades cyclin B1 to prevent its deposition, thereby inhibiting cell cycle re-entry and preventing neuronal apoptosis (Almeida et al., 2005). In the central nervous system, Cdh1 activity is required for neurogenesis and normal cortex size, as well as for the terminal differentiation of cortical neurons (Delgado-Esteban et al., 2013). Cdh1 depletion led to p53-mediated apoptosis and a decrease in the number of neurons. Whether MAD2B, an inhibitor of APC/Cdh1, is involved in the regulation of learning and memory is worth pursuing.

### RNA-seq suggested several changes in signaling pathways and gene expression profiles that are conducive to synaptic plasticity and learning and memory

*MAD2B* was conditionally knocked out in the forebrain pyramidal neurons. Therefore, we first performed RNA-seq to explore the molecular expression changes after *MAD2B* cKO in the whole hippocampus and the cerebral cortex. The extracellular matrix presented in the GO cellular component analysis of the hippocampus is involved in the regulation of the structure and function of the dendritic spine (Dityatev et al., 2010). Intracellular organelles, for example, the Golgi apparatus, are necessary for the formation of synaptic vesicles and thus play a dual role in synaptogenesis and neurotransmission (Götz et al., 2021). The cellular component analysis of the cerebral cortex revealed that DEGs were mainly involved in pre- and postsynaptic components. According to the KEGG pathway enrichment of the hippocampus, the relaxin signaling pathway has been linked to long-term spatial memory in the MWM and T maze tests (Nategh et al., 2015; Albert-Gasco et al., 2017). Other molecules, such as cell adhesion molecules, in the nervous system play an important role in synapse formation regulation (Washbourne et al., 2004), glutamate and GABA receptor trafficking (Keable et al., 2020), homeostatic synaptic plasticity (Thalhammer and Cingolani, 2014), and LTP (Wu et al., 2019). The KEGG pathway enrichment results of DEGs involving the cerebral cortical mRNA included not only glucose metabolism but also the ErbB signaling pathway, the HIF-1 signaling pathway, and LTP, all of which are related to learning and memory (Kaphzan et al., 2012; Dong et al., 2017; Ajoy et al., 2021).

We further found several DEGs related to synaptic plasticity and learning and memory ability in the hippocampus and cerebral cortex. RNA-seq and qRT-PCR results demonstrated that *Rab3b, Atf4, and Cort* were downregulated in the hippocampus and the cerebral cortex. *Rab3b*, which is a synaptic vesicle protein, is highly enriched in inhibitory synapses in the CA1 region. *Rab3b* KO mice have been reported to display selective enhancement in reversal learning (Tsetsenis et al., 2011). *Atf4*, also known as CREB2, is a synaptic plasticity feedback regulator associated with LTP (Amar et al., 2021). In addition, *Atf4* is involved in GABABR internalization and regulates neuronal excitability (Corona et al., 2018). *Cort*, a neuropeptide of the somatostatin family, has many of the same pharmacological and functional properties as somatostatin, including the ability to suppress neuronal activity. *Cort* overexpression is associated with impaired long-term spatial memory and synaptic plasticity modulation especially in aging-related cognitive deficits (Tallent et al., 2005). In addition to these downregulated genes, some genes, such as *Mthfr* in the hippocampus and *Camk2a* in the cerebral cortex, were upregulated. The severe deficiency of *Mthfr* in embryonic stem cells in pregnant mice might cause short-term memory impairment in their 3-week-old male *Mthfr*^+/+^ mice offspring as a result of apoptosis or altered choline metabolism in the hippocampus (Jadavji et al., 2015; Bahous et al., 2017). *Camk2a* is highly expressed in the forebrain, especially on the postsynaptic densities of excitatory synapses, and is related to spatial memory (Silva et al., 1992). The Ca^2+^-CaM–CaMKII signaling cascade has been well-established to be the first reaction necessary for LTP induction (Lisman et al., 2012). The expression changes of these genes and signaling pathways implied that the learning and memory ability of the *MAD2B* cKO mice may be improved.

### The *MAD2B* cKO mice showed enhanced hippocampus-dependent learning and memory

We then explored whether depleting the *MAD2B* gene in *Camk2a*-positive neurons affected learning and memory in mice. The *MAD2B* cKO mice showed normal motor coordination in the accelerating rotarod test and locomotion in the open field test. Moreover, *MAD2B* cKO did not influence anxiety levels. However, in the MWM, the escape latency and length of cKO mice on the fifth day of the training period were less than those of the WT mice. On the probe test day, although the total time in the target quadrant did not differ between the two groups, the *MAD2B* cKO mice showed increased times of crossing the location of the platform. Thus, the results of MWM indicated slightly improved spatial learning and memory of the *MAD2B* cKO mice.

To further verify the role of MAD2B in learning and memory, we then performed NORT and CFC. In the NORT, which is a hippocampus-dependent memory recognition test based on the instinctive responses of mice toward novel objects (Wu et al., 2020), the *MAD2B* cKO mice were more biased toward the novel object when tested an hour after the familiarization stage, indicating improved short-term memory than the WT mice. Both the formation and extinction of CFC are related to the hippocampus (Izquierdo et al., 2016; Lacagnina et al., 2019; Kim and Cho, 2020). In the CFC experiment, the *MAD2B* cKO mice showed normal fear acquisition and long-term fear memory with increased robustness. Therefore, these results indicated that *MAD2B* cKO in *Camk2a*-specific neurons did not impair swimming speed and fear memory acquisition but enhanced hippocampus-dependent learning and memory abilities.

### Numbers of neurons and neurogenesis in the hippocampus of the *MAD2B* cKO mice

Previous studies have further discovered that learning and memory abilities, especially spatial learning, are correlated with the number of neurons in the CA1 area (Zhang et al., 2019; Arroyo-García et al., 2021). If neuronal survival in the hippocampal CA1 subarea is improved, cognitive abilities will be enhanced (Hei et al., 2019). Therefore, we examined the numbers of neurons, astrocytes, and microglia in the hippocampus and cerebral cortex to explore the mechanism of enhanced learning and memory in the *MAD2B* cKO mice. Our results indicated that *MAD2B* cKO selectively increased the number of neurons in the cerebral cortex and the CA1 region. However, we found no differences in the CA3 and DG regions between the two groups. Moreover, we discovered that *MAD2B* cKO did not affect the numbers of microglial cells or astrocytes. These results were consistent with previous findings showing that MAD2B is mainly located in neurons in the central nervous system (Meng et al., 2012). Thus, *MAD2B* cKO mainly affected the number of neurons.

MAD2B is known as a component of the mitotic spindle assembly checkpoint. In a high-glucose environment, the increased expression of MAD2B in primary cultured neurons induces cell cycle re-entry (Meng et al., 2014). Therefore, the stable expression of MAD2B plays an important role in maintaining neuronal cell cycle homeostasis. The neural stem cells present in the DG area of the hippocampus play an important role in complex learning and memory ability (Zhao et al., 2008). We then wondered if the enhancement in learning and memory in the *MAD2B* cKO mice was related to the neurogenesis in the DG area. Our analysis showed that the number of DCX^+^ neurons in the DG of the hippocampus had increased. This result suggested that *MAD2B* cKO enhanced neurogenesis in the DG and thus played a potential role in increasing learning and memory.

### Dendritic branches, dendritic spines, and synapses of neurons changed to facilitate synaptic plasticity after *MAD2B* cKO

The pathogenesis of cognitive disorders is influenced not only by neuron number but also by synaptic plasticity. MAD2B regulates the APC/Cdh1 activity, which plays an important role in regulating synaptic plasticity in the mammalian brain (Huang et al., 2015). APC/Cdh1 mediates the EphA4-dependent downregulation of AMPA receptors in homeostatic plasticity and is required for associative fear memory and long-term potentiation in the amygdala of adult mice (Pick et al., 2012). Late-phase LTP was found to be impaired in mice with Cdh1 cKO mediated by the Nse-cre driver (Li et al., 2008; Pick et al., 2013). Changes in dendritic branches and dendritic spine number and shape are important bases of synaptic plasticity regulation. In many neurodegenerative diseases, cognitive decline is frequently accompanied by a reduction in dendritic branches (Mehder et al., 2020, 2021; Mendell et al., 2020). High numbers of dendritic branches are usually associated with memory improvement (Liu et al., 2008; Orellana et al., 2018). In the present study, Golgi staining showed that although the *MAD2B* cKO mice had a similar number of dendritic branches as the WT group, they had more dendritic spines in CA1 neurons than the WT mice. The number of mushroom- and long-like spines was also increased in the *MAD2B* cKO mice. Recent studies on spine plasticity have demonstrated that modifications to the shapes of the spine neck and head may be cellular indicators of learning and memory. An enlarged spine head is indicative of increased contact surfaces with its synaptic partner, numerous AMPARs on the postsynaptic membrane, and the elevated amplitude of AMPAR-related currents (Harris and Stevens, 1989; Schikorski and Stevens, 1997; Takumi et al., 1999; Matsuzaki et al., 2001). A shortened and widened spine neck facilitates the transportation of information between dendrites and the spine (Tonnesen et al., 2014). Therefore, the increased number of mushroom-like dendritic spines was one of the structural foundations of memory enhancement in the *MAD2B* cKO mice. Some researchers have divided dendritic spines into two categories: small spines (filopodial like- and thin-/long- spines) and large spines (stubby- and mushroom-like spines). Small spines are more changeable than large ones, whereas large spines are more stable than small ones. In the learning process, small spines have the potential to develop into large spines (Parnass et al., 2000; Grutzendler et al., 2002; Trachtenberg et al., 2002; Kasai et al., 2003). In our study, the *MAD2B* cKO mice had numerous long dendritic spines, which indicated increased learning plasticity ability.

In addition to the structural complexities of dendritic branches and spines, different types of synapses play important roles in synaptic plasticity. Excitatory and inhibitory synapses release different neurotransmitters, inducing LTP or LTD. Although inhibitory synapses target dendritic shafts (Chen et al., 2012) and excitatory synapses target dendritic spines (Aoto et al., 2007), the majority of glutamatergic excitatory synapses are located on dendritic spines (Berry and Nedivi, 2017), and GABAergic inhibitory synapses are located on dendritic shafts (Wierenga et al., 2008). The state of some shaft synapses can be changed. When some stimuli induce LTP, shaft synapses can gradually change into spine synapses, increasing AMPARs (Xu et al., 2020). Our results showed that the *MAD2B* cKO mice had more spine synapses and fewer shaft synapses in the CA1 area than the WT mice. Therefore, in the *MAD2B* cKO mice, the presence of numerous spine synapses was indicative of numerous excitatory spines, whereas the presence of few shaft synapses was indicative of few inhibitory spines (Lee et al., 2021), resulting in improved learning and memory.

In conclusion, we determined the role of MAD2B in learning and memory by knocking out *MAD2B* in *Camk2a*-specific neurons. We demonstrated that the *MAD2B* cKO mice exhibited changes in the signaling pathways and genes associated with synaptic plasticity, although in the present study there was a relative limitation as we used the whole cerebral cortex and hippocampus. The *MAD2B* cKO mice had enhanced hippocampus-dependent learning and memory. The cKO of endogenous *MAD2B* in *Camk2a*-specific neurons increased the number of neurons in the CA1 and cerebral cortex, enhanced neurogenesis in the DG, and promoted the branching of dendrites and the growth of dendritic spines. All of these factors are conducive to improved learning and memory. However, numerous aspects of the *MAD2B* cKO mice, such as the influence and mechanisms of hippocampus-dependent memory in juvenile or senescent *MAD2B* cKO mice, remain unknown. These prompt us to explore further functions of *MAD2B* in learning and memory.

## Data availability statement

The datasets presented in this study can be found in online repositories. The name of the repository and accession number can be found below: National Center for Biotechnology Information (NCBI) BioProject, https://www.ncbi.nlm.nih.gov/bioproject/, PRJNA866907.

## Ethics statement

The animal study was reviewed and approved by Huazhong University of Science and Technology Ethics Committee for Care and Use of Laboratory Animals.

## Author contributions

LC performed the experiments with assistance from YS and KZ. YX analysed the data. CZ and XM supervised the project. LC, XM, and CZ wrote the manuscript. All authors contributed to the article and approved the submitted version.

## Funding

This work was supported financially by grants from the National Natural Science Foundation of China (Grant Nos. 81974162 and 81671066).

## Conflict of interest

The authors declare that the research was conducted in the absence of any commercial or financial relationships that could be construed as a potential conflict of interest.

## Publisher's note

All claims expressed in this article are solely those of the authors and do not necessarily represent those of their affiliated organizations, or those of the publisher, the editors and the reviewers. Any product that may be evaluated in this article, or claim that may be made by its manufacturer, is not guaranteed or endorsed by the publisher.
