# Supplementary material for: Conditional deletion of MAD2B in forebrain neurons enhances hippocampus-dependent learning and memory in mice
Source: Front Cell Neurosci. 2022 Sep 23;16:956029. doi: 10.3389/fncel.2022.956029 (PMC9538151; doi:10.3389/fncel.2022.956029)
Supplement: Supplementary file 1 [file Data_Sheet_1.docx]

**Supplementary material**

Method to generate MAD2B^flox/flox^ mice:

The bacterial artificial chromosome (BAC) clones containing the target gene MAD2B were purchased from Sanger Institute (UK). The embryonic stem (ES) cell targeting vector was constructed by ET-clone method. The vector contains 5.95 KB 5 'homology arm, 950bp flox region, PGK-Neo-polyA, 3.1 KB 3' homology arm and MC1-TK-polyA negative screening marker. After linearization, the vector was electrically transferred to C57/129 hybrid ES cells. After drug screening of G418 and Ganc, resistant clones were obtained. The positive clones with correct homologous recombination were identified by long fragment PCR. The positive ES cell clones were amplified and injected into the blastocysts of C57BL/6J mice to obtain chimeric mice. A high proportion of chimeric mice were mated with Flp C57BL/6J mice to obtain MAD2B^flox/flox^ mice.


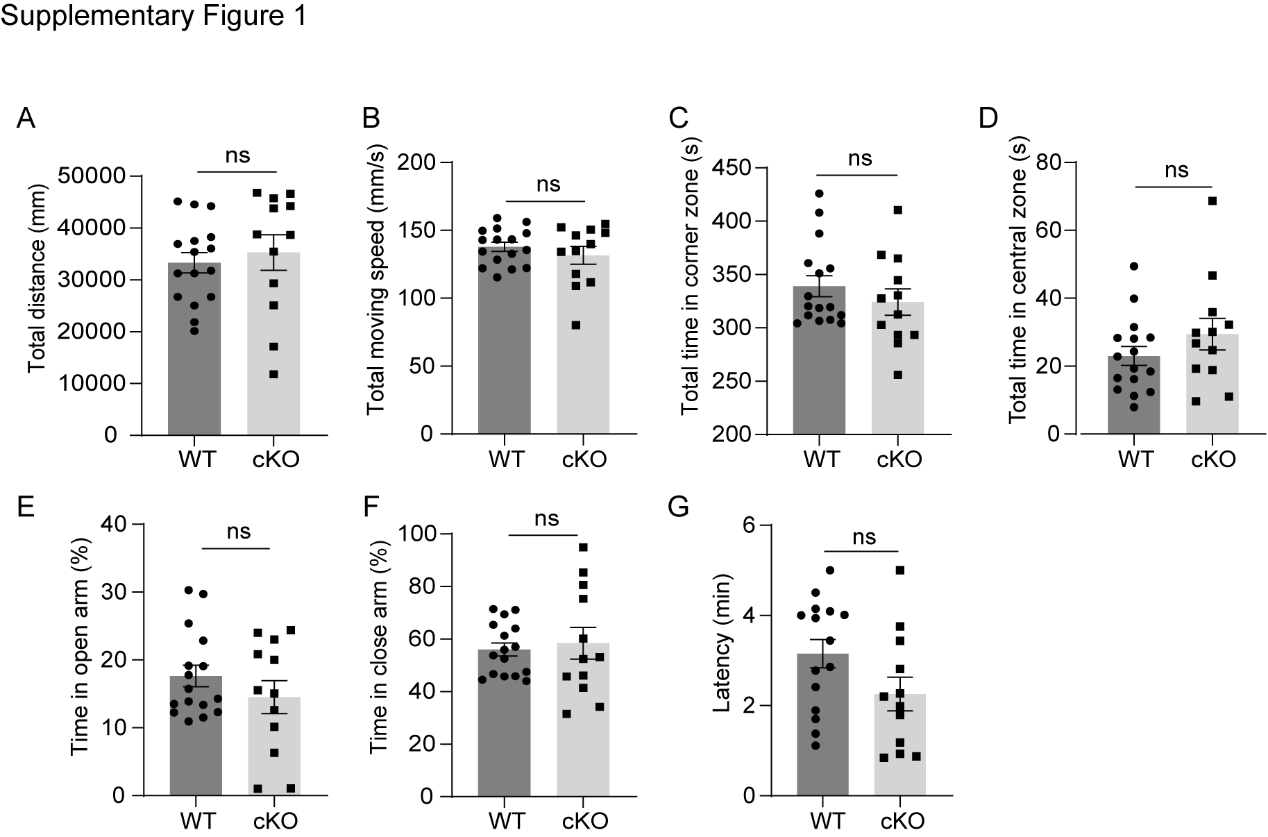


Supplementary Figure 1

(**A-D**) Total distance (*P*=0.5979, *t*=0.5340), total moving speed (*P*=0.3712, *t*=0.9100), time in the corner zone (*P*=0.3481, *t*=0.9555), and time in the central zone (*P*=0.2191, *t*=1.259) in open field test showed no significant difference between WT and cKO groups, unpaired two-tailed *t*-tests. **(E-F)** Time in open arms (*P*=0.2727, *t*=1.121) and close arms (*P*=0.7198, Welch-corrected *t*=0.3657) of MAD2B cKO mice was similar to that of the WT group in the elevated plus maze. *n*=16 in the WT group, and *n*=12 in the cKO group. (**G**) Motor coordination verified by falling latency of rotarod test showed cKO didn’t influence the locomotor ability. (*P*=0.0787, *t*=1.833), unpaired two-tailed *t*-test, *n*=15 in WT group, *n*=12 in cKO group. “ns” indicates no significance. All data are displayed as means ± s.e.m.


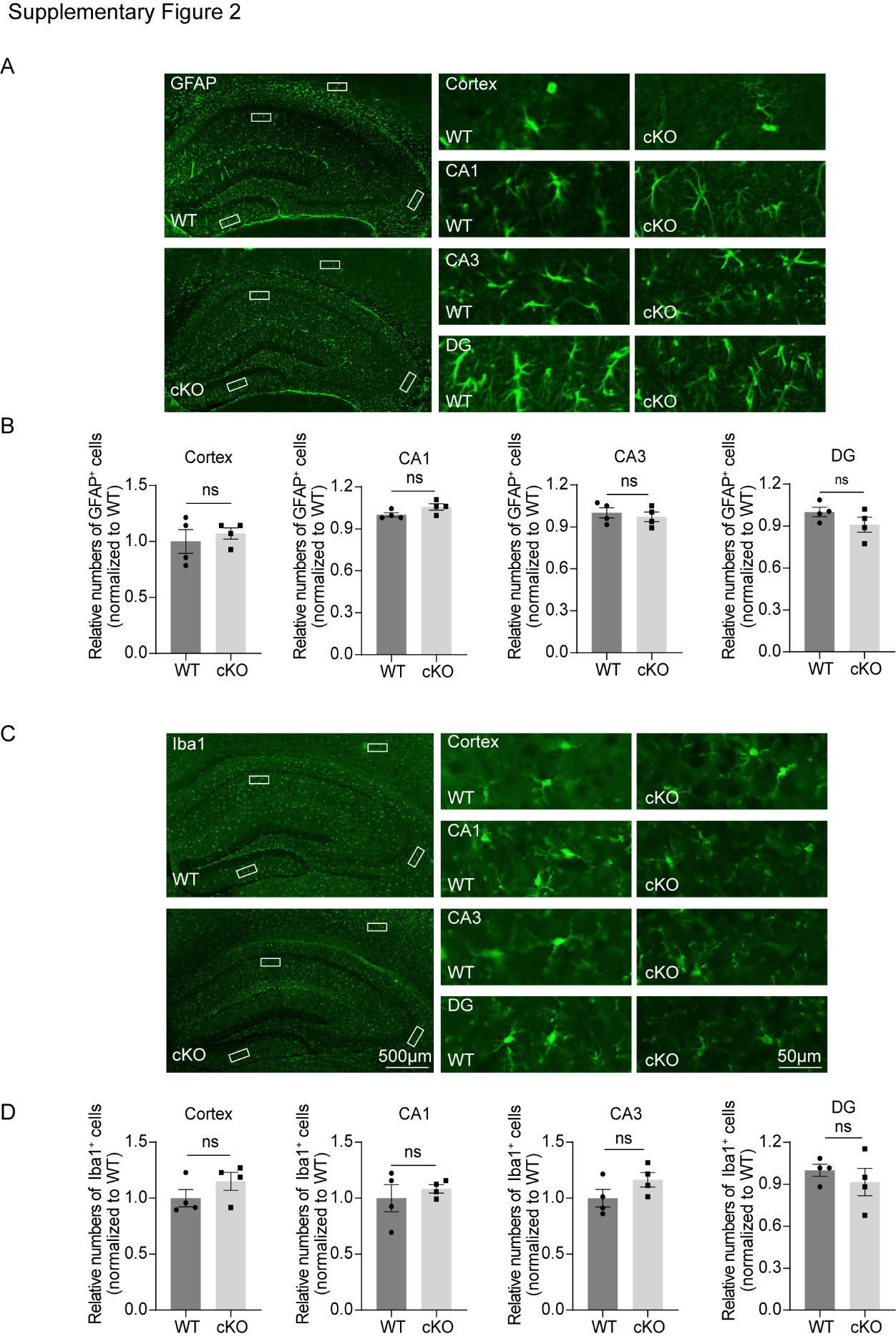


Supplementary Figure 2

**(A)** Representative fluorescent images of GFAP in the hippocampus and cortex. The dashed box labels the representative areas of the cortex, CA1, CA3, and DG. The scale bar is 500 μm. Representative enlarged images of these areas are shown on the right, and the scale bar is 50 μm. **(B)** Quantification analysis of GFAP^+^ cells showed there was no difference between WT and cKO groups in the number of astrocytes in cortex (*P*=0.5628, *t*=0.6124) and hippocampus (CA1, *P*=0.0881, *t*=2.035, CA3, *P*=0.6015, *t*=0.5510, DG, *P*=0.1928, *t*=1.446). *n*=4, unpaired two-tailed *t*-test. **(C)** Representative fluorescent images of Iba-1 in the hippocampus and cortex. The dashed box labels the representative areas of the cortex, CA1, CA3, and DG. The scale bar is 500 μm. Representative enlarged images of these areas are shown on the right, and the scale bar is 50 μm. **(D)** Quantification analysis of Iba-1^+^ showed there was no difference between WT and cKO groups in the number of microglia in the cortex (*P*=0.2237, *t*=1.357) and hippocampus (CA1, *P*=0.5353, *t*=0.6575, CA3, *P*=0.1580, *t*=1.612, DG, *P*=0.4575, *t*=0.7939). *n*=4, unpaired two-tailed *t*-test. “ns” indicates no significance. All data are displayed as means ± s.e.m.
